# Supplementary material for: The Virucidal Effect of the Chlorination of Water at the Initial Phase of Disinfection May Be Underestimated If Contact Time Calculations Are Used
Source: Pathogens. 2023 Oct 3;12(10):1216. doi: 10.3390/pathogens12101216 (PMC10609707; doi:10.3390/pathogens12101216)
Supplement: Supplementary file 1 [file pathogens-12-01216-s001.zip › pathogens-2632811-supplementary.pdf]

Supplementary

Table S1. Analysis of the kinetic of chlorine adsorption in the two cell media by addition of different indicated amounts of chlorine. Media substrates were diluted 1:1000 ratio before tested.

NaOCl (VOL) in 5 l	Media	(mg/l)	Time (min)								
			**0**	**2**	**6**	**14**	**18**	**53**	**57**	**123**	**127**
**6A (50 µl)**	**Ultra-filtrated Water**	Free chlorine	0.98	0.98		0.82		0.69		0.64	
	**MEM substrate**	Free chlorine	0.98		0.39		0.31		0.12		0.08
	**M119 substrate**	Free chlorine	0.98		0.27		0.23		0.11		0.06
**6B (60 µl)**	**Ultra-filtrated Water**	Free chlorine	1.31	1.22		1.08		1.05		1.00	
	**MEM substrate**	Free chlorine	1.32		0.60		0.28		0.18		0.12
	**M119 substrate**	Free chlorine	1.29		0.54		0.28		0.19		0.13
**6C (65 µl)**	**Ultra-filtrated Water**	Free chlorine	0.65	0.57					0.48	0.46	
	**MEM substrate**	Free chlorine	0,72		0,07	0,07	0,06			0,05	
	**M119 substrate**	Free chlorine	0,71		0,14		0,08	0,06			0,05

Table S2. Analysis of the comcentration of chlorine dioxide by the two different cell media by addition of 0.17 mg ClO_2/l. media substrates were diluted 1:1000 ratio before tested.

Media	Disinfectant residual (mg/l)	Time (min)												
		**0**	**3**	**6**	**10**	**27**	**31**	**35**	**91**	**95**	**99**	**132**	**135**	**140**
**M119 substrate**	ClO_2	0.16			0.136			0.101			0.074			0.075
**MEM substrate**	ClO_2	0.16		0.132			0.129			0.091			0.077	
**Ultra-filtrated Water**	ClO_2	0.17	0.135			0.120			0.103			0.090		

Table S3. The total Chlorine dioxide and Chlorine concentrations calculated in water suspension with echovirus 30, simian rotavirus and adenovirus type 2 at 0, 2,10, 30, 60, and 120 minutes. The mean transmittance (254nm) was 91.4% and TOC was 2.4 mg/l before the start of the experiments. The pH and temperatures are given at the beginning and end of the experiments.

		Free chlorine after treatment (minutes)									
Virus	Disinfectants (mg/l)	pH	temp	0	2	3	6	10	30	60	120
Echovirus exp1	Chlorine dioxide	6.7-6.7	12C-21C	0.53	0.20			0.26	0.17	0.11	0.07
	Chlorine	6.7-6.8	12C-16C	1.31	0.37	0.17	0.16	0.18	0.15	0.	0.12
Echovirus exp2	Chlorine dioxide	6.7-6.7	9C-16C	0.53	0.26			0.18	0.17	0.13	0.09
	Chlorine	6.8-6.8	9C-16C	1.31	0.5			0.29	0.21	0.11	0.11
Rotavirus Exp1	Chlorine dioxide	6.7-6.8	9C-19C	0.49		0.25		0.09		0.09	<0.03
	Chlorine	6.7-6.8	9C-19C	1.2	0.53			0.41	0.25	0.14	0.10
Rotavirus exp2	Chlorine dioxide	6.7-6.7	9C-20C	0.52		0.16		0.29	0.19	0.12	0.06
	Chlorine	6.7-6.7	9C-20C	1.13	0.44			0.31	0.18	0.14	0.11
Adenovirus exp1	Chlorine dioxide	6.7-6.7	8C-17C	0.47	0.25			0.22	0.16	0.10	<0.03
	Chlorine	6.7-6.9	8C-17C	1.31	0.55			0.32	0.15	0.10	0.08
Adenovirus Exp2	Chlorine dioxide	6.7-6.7	8C-19C	0.56	0.35				0.21	0.21	0.08
	Chlorine	6.7-6.8	7C-13C	1.31	0.35			0.21	0.14	0.12	0.12

Table S4. Primers and probes used for qPCR detection of E30. R. and HAdV2.

Primers	Primer Sequences	Reference
E30-forward	TCCTCCGGCCCCTGAATGCG	(Wang et al., 2020b)
E30-reverse1	ACCGGATGGCCAATCCAA	(Wang et al., 2020b)
E30-reverse2	ATGTCACCATAAGCAGCCA	(Wang et al., 2020b)
E30-probe	CGGAACCGACTACTTTGGGTGIC	(Wang et al., 2020b)
RV SA11-forward	ACC ATC TWC ACR TRA CCC TCT ATG AGA	(Zeng et al., 2008)
RV SA11-reverse	GGT CAC ATA ACG CCC CTA TAG C	(Zeng et al., 2008)
RV SA11-probe	[FAM] AGT TAA AAG CTA ACA CTG TCA AA [MGBEQ]	(Zeng et al., 2008)
HAdV2-forward	GCCACGGTGGGGTTTCTAAACTT	(Wang et al., 2018)
HAdV2-reverse	GCCCCAGTGGTCTTACATGCACATC	(Wang et al., 2018)
HAdV2-probe	TGCACCAGACCCGGGCTCAGGTACTCCGA	(Wang et al., 2018)
